# The Influence of Explainable Artificial Intelligence and Mental Health Literacy on Professional Psychological Help-Seeking Behavior: The Mediating Role of Psychological Problem Cognition

**DOI:** 10.3390/bs15121646

**Published:** 2025-11-30

**Authors:** Weijun Wang, Shijie Gao, Qian Chen, Shihao Ma

**Affiliations:** 1 Key Laboratory of the Ministry of Education on Adolescent CyberPsychology and Behavior, Central China Normal University, Wuhan 430079, China; wangwj@ccnu.edu.cn (W.W.); cecilia_gsj@163.com (S.G.); quorrachen07@mails.ccnu.edu.cn (Q.C.); 2 School of Psychology, Central China Normal University, Wuhan 430079, China

**Keywords:** explainable artificial intelligence, mental health literacy, professional psychological help-seeking behavior, psychological problem cognition

## Abstract

Approximately 970 million people worldwide suffer from mental disorders, yet over 60% do not seek professional help. Key barriers include low mental health literacy (MHL), insufficient awareness of psychological issues, and the overwhelming and non-transparent nature of information retrieval tools. Explainable artificial intelligence (XAI) can provide transparent and personalized feedback; however, its unique contribution to facilitating professional psychological help-seeking behavior remains unclear. Two randomized scenario-based experiments were conducted. Experiment 1 (*n* = 168) examined the effect of XAI on professional psychological help-seeking behavior. Experiment 2 (*n* = 178), utilizing a virtual simulation program based on AI search, tested the interaction between XAI and MHL and the mediating effect of psychological problem recognition. Both XAI and MHL significantly enhanced participants’ psychological problem recognition and professional psychological help-seeking behavior. A significant interaction was observed; XAI had a stronger facilitative effect on psychological problem recognition and professional psychological help-seeking behavior, particularly among participants with lower MHL. Furthermore, psychological problem recognition mediated the effects of both XAI and MHL on professional psychological help-seeking behavior. XAI can compensate for low MHL by reshaping individuals’ psychological problem recognition, thereby promoting the utilization of professional mental health services.

## 1. Introduction

The World Health Organization reported that approximately 970 million people worldwide suffer from mental disorders, including anxiety and depression, but due to underdiagnosis and stigma, the actual prevalence may be significantly underestimated. Studies show that over 60% of individuals with mental disorders do not seek professional help (WHO, 2022). This phenomenon is closely linked to individuals’ insufficient recognition of their psychological problems. Many individuals fail to recognize the nature of their symptoms, underestimate the severity of their condition, or hold negative attitudes toward professional treatment, such as low expectations for therapy or fear of stigma, which ultimately leads to delayed or avoided help-seeking (Billings et al., 2021; Doll et al., 2021; da Conceição et al., 2024).

The widespread use of the internet has created new opportunities to enhance individuals’ recognition of mental health issues. Online search engines have become important tools for obtaining mental health information, enabling individuals to independently access relevant resources for self-help (Bizzotto et al., 2023; Sherman et al., 2021). Through online searches, individuals can more easily identify their problems and develop coping strategies, thereby increasing the likelihood of seeking professional psychological help (Gulliver et al., 2010).

However, traditional search engines are limited by low efficiency in filtering information, an overwhelming amount of content, and challenges in verifying the reliability of sources, making it difficult for users to access trustworthy and actionable knowledge.

The development of artificial intelligence (AI) technologies offers new possibilities for addressing these challenges. AI-powered search engines, with their powerful capabilities in information integration and generation, overcome many limitations of traditional tools by collecting, processing, and presenting information more efficiently and accurately to meet individuals’ informational needs (X. K. Song et al., 2023). However, the application of AI algorithms still suffers from a lack of transparency (Koutsouleris et al., 2022). AI’s “black box” nature, characterized by opaque decision-making processes, limits users’ ability to trace how recommendations are generated or assess their validity (Y. Zhang et al., 2022). Research has shown that individuals are generally reluctant to adopt technologies that lack explainability, are difficult to use, or are perceived as untrustworthy (Holzinger et al., 2017). Therefore, in the domain of mental health, the development of explainable artificial intelligence (XAI) is of critical importance.

Explainable artificial intelligence (XAI) refers to intelligent systems that provide clear and transparent information about their decision-making processes, enabling users to understand how information is processed and how recommendations are generated. Enhancing the explainability of AI not only improves users’ trust in AI-assisted decision-making but may also influence their behavioral choices (Barredo Arrieta et al., 2020; X.-H. Li et al., 2020). Previous studies have demonstrated the positive impact of XAI in domains such as healthcare and high-stakes decision-making (Markus et al., 2021; Leichtmann et al., 2023). Given that professional psychological help-seeking can also be regarded as a form of health-related decision-making (Gao, 2014), an important question arises: Can XAI similarly promote individuals’ professional psychological help-seeking behavior in the mental health context? And through what mechanisms might such effects occur? These issues warrant further investigation.

In addition, mental health literacy, an individual’s cognitive ability to recognize and understand mental health issues, has been shown to positively correlate with professional psychological help-seeking behavior (Baklola et al., 2024; J. Yang et al., 2023). However, it remains unclear whether and how individuals’ help-seeking behavior might change when mental health literacy interacts with explainable AI as an external informational resource. Does the impact of XAI vary depending on an individual’s level of mental health literacy? These questions have not yet been systematically explored.

In summary, this study aims to investigate the impact of explainable artificial intelligence (XAI) on professional psychological help-seeking behavior in the context of health information search. Specifically, it seeks to answer whether XAI can effectively promote individuals’ intention to seek professional psychological help. Furthermore, the study explores the interaction between mental health literacy and XAI, aiming to reveal whether the influence of XAI on help-seeking behavior differs across individuals with varying levels of mental health literacy and to uncover the underlying mechanisms. This research contributes not only to expanding the application of XAI in the field of mental health but also provides theoretical support and practical guidance for the development of human-centered AI technologies.

## 2. Related Work

### 2.1. Professional Psychological Help-Seeking Behavior and Its Predictors

Professional psychological help-seeking behavior refers to the decision-making process in which individuals experiencing psychological distress seek assistance from qualified professionals or institutions (either online or offline) for psychological support, including diagnosis, counseling, emotional disclosure, and treatment (Rickwood et al., 2005; L. X. Liu & Yang, 2006; Jiang & Xia, 2006). In essence, professional psychological help-seeking is a type of health-related decision-making behavior (Gao, 2014), and its occurrence is influenced by individuals’ cognitive processes.

According to the Health Belief Model (HBM), the adoption of health behaviors requires adequate cognitive awareness. When individuals can accurately recognize a health issue and perceive its severity and personal relevance, health-related actions are more likely to be triggered (Rosenstock et al., 1988). The model further proposes that individuals’ willingness to seek medical services is influenced by risk perception and behavioral evaluation. Risk perception comprises perceived susceptibility and perceived severity, while behavioral evaluation involves perceived benefits and perceived barriers.

In the context of mental health, studies have shown that the recognition of mental health problems, the perceived severity of these problems, and beliefs in the effectiveness of treatment are key cognitive factors influencing help-seeking behavior (Billings et al., 2021; Doll et al., 2021; Bu et al., 2023). Compared to general physical health issues, psychological disorders often present more complex and ambiguous symptoms. In addition to emotional distress, individuals may experience somatic symptoms such as headaches, back pain, or chest pain (X. J. Wang & Lv, 2010), which may be misattributed to physical illness or external stress. Such misrecognition can hinder the initiation of professional psychological help-seeking behavior (Pátková Daňsová et al., 2024; Fu et al., 2024).

Moreover, individuals often underestimate the severity of mental health issues. For instance, Spiers et al. (2017) conducted semi-structured interviews with 47 general practitioners and found that their low recognition of the seriousness of mental health problems led them to believe that professional help was unnecessary.

Belief-related barriers can also impede help-seeking (Rickwood et al., 2005). Individuals may hesitate to seek help due to doubts about the effectiveness of treatment or a lack of trust in mental health professionals (Wu et al., 2010; Y. Chen et al., 2010; Eisenberg et al., 2007). Stigma represents another major barrier (Bu et al., 2023; Doll et al., 2021). Studies have shown that internalized stigma surrounding mental illness can significantly reduce individuals’ willingness to seek help (X. D. Yang et al., 2023). However, reducing stigma has been shown to significantly improve help-seeking behavior. For example, in an experimental study on stigma intervention, participants in the intervention group reported significantly greater willingness to seek professional help (da Conceição et al., 2024). Taken together, these findings suggest that improving individuals’ recognition of mental health problems is a crucial prerequisite for promoting professional psychological help-seeking behavior.

How, then, can individuals’ recognition of mental health problems be effectively improved? Scholars suggest that information plays a crucial role in this process. Previous studies have shown that information can significantly influence individuals’ psychological help-seeking behavior, particularly when cognitive awareness is insufficient. In such cases, access to relevant information can compensate for the lack of knowledge, helping individuals more accurately identify their symptoms and make informed help-seeking decisions (J. Yang et al., 2023; Luo, 2021). Some researchers also argue that professional psychological services can be viewed as special goods from a marketing perspective. Thus, by optimizing information dissemination strategies, it may be possible to promote professional psychological help-seeking behavior more effectively (J. Y. Li et al., 2023; Q. Li & Gao, 2011).

From a health communication standpoint, X. D. Yang et al. (2023) found that the frequency of information-seeking significantly influenced public health beliefs regarding mental health, with perceived benefits emerging as the strongest predictor within the health belief framework. Additionally, exposure to media content related to professional psychological services, such as mental health advertisements, has been shown to improve attitudes toward psychotherapy and increase treatment-seeking willingness (X. Song et al., 2024).

The widespread use of the internet has facilitated easier access to health-related information. In the context of mental health, where stigma often inhibits help-seeking, the anonymity of online platforms reduces psychological barriers, making individuals more inclined to seek mental health information online (Bizzotto et al., 2023; Gulliver et al., 2010; Rice et al., 2014). Among online information-seeking methods, search engines are the most commonly used. Statistics show that over 65% of internet users worldwide use search engines to look up health information (Fox, 2009; Hou & Sun, 2015). Through searching, browsing, evaluating, and selecting mental health information, individuals are better positioned to decide whether to pursue professional psychological help (Luo, 2021).

Therefore, this study situates professional psychological help-seeking behavior within the context of health information search and conceptualizes it as a sequential process of “information-cognition-decision.” It aims to explore how information can influence help-seeking behavior by reshaping individuals’ recognition of mental health problems. Specifically, the study seeks to answer the following central question: In the context of health information search, how can the optimization of information content effectively promote individuals’ professional psychological help-seeking behavior?

### 2.2. XAI and Professional Psychological Help-Seeking Behavior

The public increasingly relies on the internet to obtain mental health information and assess whether professional help is needed (Bizzotto et al., 2023; Morahan-Martin, 2004), Scholars suggest that such health information-seeking behavior generally has positive effects. When equipped with adequate information about their health status, individuals are more likely to make informed and rational treatment decisions (X. Jia et al., 2021). However, the overabundance of online information can lead to information overload. This makes it difficult for individuals to effectively filter out useful and relevant content (Zhou et al., 2024), potentially undermining the benefits of information seeking.

The development of generative artificial intelligence (GAI) offers new possibilities for addressing this issue. Increasingly, GAI is being embedded into mainstream internet search engines, such as Microsoft’s New Bing and Google’s Bard. This trend has also spurred the emergence of AI-native search engines, including Meta’s Meta AI Search and Perplexity AI. These advanced systems provide not only interactive, dialogue-based responses to user queries but also real-time assistance with information retrieval and consultation. By actively offering search suggestions, they guide users toward more accurately locating the information they need (Schneider et al., 2023).

Despite these advancements, the “black-box” nature of AI algorithms—characterized by opaque and uncontrollable information processing—limits their practical utility (X. K. Song et al., 2023). Research indicates that people are often reluctant to adopt technologies that are difficult to understand, operate, or trust (Holzinger et al., 2017). Consequently, enhancing the explainability of AI systems, known as Explainable Artificial Intelligence (XAI), has become a critical direction for improving the effectiveness of health information search (Guidotti et al., 2019).

XAI refers to intelligent systems capable of providing clear and transparent information about their decision-making processes. This helps users understand how information is processed and how recommendations are generated (Barredo Arrieta et al., 2020; X.-H. Li et al., 2020). Unlike traditional “black-box” models, XAI not only ensures the accuracy of search results but also presents the underlying information filtering logic, recommendation rationale, and risk assessments in an understandable manner. This transparency enhances users’ trust in and willingness to use the information provided (Scharowski et al., 2023).

Previous studies have demonstrated the positive impact of XAI on individual decision-making, particularly in the fields of medical and high-risk decisions. For instance, Markus et al. (2021) found that explainable modeling in the healthcare context can improve the perceived credibility of AI systems, though further evidence is needed to verify its practical impact. In another study, Leichtmann et al. (2023) assessed how explanation methods influence AI-assisted decision-making. Participants engaged in a virtual mushroom-picking task, using an AI application to categorize mushrooms as edible or poisonous. The results showed that groups receiving explanation-enhanced feedback performed significantly better than those without explanations.

As professional psychological help-seeking behavior involves both medical and high-risk decision-making, XAI is likely to exert a similar influence. Moreover, as previously discussed, help-seeking can be understood as a sequential process involving information acquisition, cognitive recognition, and decision-making. Therefore, XAI may facilitate professional help-seeking by enhancing users’ understanding of psychological health information throughout this process.

### 2.3. Mental Health Literacy and Professional Psychological Help-Seeking Behavior

As previously discussed, cognitive factors are critical determinants of professional psychological help-seeking behavior. Mental health literacy—defined as the knowledge and beliefs that aid in the recognition, management, and prevention of mental disorders (Jorm et al., 1997)—reflects an individual’s internal cognitive capacity regarding mental health.

Research consistently shows a positive correlation between mental health literacy and help-seeking attitudes. Individuals with higher mental health literacy are more likely to seek professional assistance (Baklola et al., 2024; J. Yang et al., 2023). This relationship may be attributed to their enhanced ability to accurately identify psychological problems and recognize the necessity of professional intervention. Conversely, individuals with lower levels of mental health literacy tend to misinterpret symptoms, underestimate the severity of psychological issues, and may be more vulnerable to stigmatization, all of which decrease the likelihood of seeking help (Billings et al., 2021).

Empirical studies have confirmed this significant positive relationship. For example, Gorczynski et al. (2017) demonstrated the link between mental health literacy and professional help-seeking behavior. Similarly, Y. F. Jia et al. (2023) found that among university students, those with higher mental health literacy were more likely to seek help online or recommend professional services to others.

Although mental health literacy directly influences help-seeking behavior, this influence does not occur in isolation; rather, it is likely shaped by the external information environment. According to the Health Belief Model, external information—such as peer evaluations, expert advice, and online content—serves as a critical cue to action that encourages individuals to adopt health-related behaviors (Maiman & Becker, 1974; Rosenstock, 2005; Hao, 2009).

Empirical research supports this view, showing that online health information plays an essential role in shaping individuals’ health beliefs and attitudes toward help-seeking (Kealey & Berkman, 2010). As more online search engines incorporate artificial intelligence technologies, AI may become a crucial source of information that influences how individuals recognize psychological problems and decide whether to seek professional help.

XAI, as an external information factor, can influence decision-making processes through its transparency and comprehensibility. By providing clear explanations of its reasoning, XAI has the potential to promote help-seeking behavior. Meanwhile, mental health literacy, as an internal cognitive factor, provides the necessary knowledge and belief system to support such behavior. When individuals are deciding whether to seek professional psychological help, mental health literacy helps them better understand and utilize the information provided by XAI systems. Conversely, by enhancing information transparency and interpretability, XAI may reshape individuals’ knowledge and beliefs about psychological problems. This reciprocal relationship suggests that internal literacy and external information systems may interact in shaping help-seeking decisions.

However, current research rarely explores how mental health literacy interacts with informational factors to jointly influence professional psychological help-seeking behavior. Based on this, the present study will further examine the interaction between mental health literacy and explainable AI, aiming to uncover how these two factors jointly shape individuals’ decisions to seek professional psychological help.

### 2.4. The Mediating Role of Psychological Problem Cognition

What are the underlying psychological mechanisms through which XAI and mental health literacy influence professional psychological help-seeking behavior? This study proposes that recognition of psychological problems may serve as a key mediating variable in this process.

Psychological problem cognition refers to an individual’s understanding and assessment of their mental health status (Saleeby, 2000; J. Yang et al., 2023). Research has demonstrated that mental health literacy significantly impacts individuals’ recognition of psychological problems. Individuals with higher levels of mental health literacy tend to possess a more accurate and comprehensive understanding of their mental health conditions (J. Yang et al., 2023). They are typically better able to identify psychological symptoms, perceive the associated risks, and recognize the benefits of professional help. These enhanced cognitive capacities ultimately make them more likely to engage in professional psychological help-seeking behavior.

XAI may also influence individuals’ recognition of psychological problems. By clarifying the decision-making process between input and output, XAI enhances the clarity and perceived credibility of mental health information (Buçinca et al., 2020), thereby potentially improving users’ understanding of their mental health status. According to the Theory of Mind, humans tend to coordinate their actions by inferring the intentions of others (Corbetta et al., 2008). XAI simulates this process by offering explanations aligned with human cognitive frameworks. It uses understandable logic to make AI appear as a “communicative partner” rather than a cold, inhuman tool (Carter et al., 2024). This reduces users’ cognitive burden during information processing, allowing them to focus more fully on the content, and may thereby enhance their awareness and evaluation of their current mental state.

The Health Belief Model posits that recognizing one’s current psychological condition is a prerequisite for help-seeking behavior. Similarly, the Theory of Planned Behavior asserts that behavior is driven by cognition and proceeds through a process of information processing, analysis, and reasoning. Only when individuals hold a positive attitude toward the behavior, perceive social support, and believe in their ability to perform the behavior will they form the intention to act and follow through with it (Ajzen, 1991; Ji, 2023).

In mental health contexts, research confirms that individuals who avoid professional help often lack adequate recognition of their psychological problems (Billings et al., 2021). This typically stems from misjudging symptoms or underestimating their severity (Coles & Coleman, 2010; Gong & Song, 2024). An individual’s mental health literacy reflects their level of psychological problem cognition. At the same time, XAI provides clear and transparent explanatory information, helping individuals better identify and understand their mental health conditions. This enhanced recognition increases the likelihood that users will trust and follow AI recommendations, ultimately promoting professional psychological help-seeking behavior. Therefore, both mental health literacy and explainable AI may influence help-seeking behavior through the mediating effect of psychological problem cognition.

This study positions recognition of psychological problems as a mediating variable to explore how mental health literacy and explainable artificial intelligence (XAI) influence individuals’ professional psychological help-seeking behavior through their impact on psychological problem cognition. This research contributes to a deeper understanding of the internal decision-making process individuals undergo when facing mental health issues and provides a theoretical foundation and practical guidance for promoting professional psychological help-seeking behavior.

### 2.5. The Present Study

In summary, this study aims to investigate the impact of explainable artificial intelligence (XAI) and mental health literacy on professional psychological help-seeking behavior, as well as the mediating role of psychological problem cognition, through two controlled experiments. The research seeks to address the following questions:

(1) What is the effect of explainable artificial intelligence on individuals’ professional psychological help-seeking behavior?
(2) Do explainable artificial intelligence and mental health literacy interact to produce differential effects on psychological problem cognition and help-seeking behavior?
(3) Does psychological problem cognition mediate the effects of explainable artificial intelligence and mental health literacy on professional psychological help-seeking behavior?

## 3. Study 1

This study aims to investigate the impact of the explanation provided by an AI search engine on individuals’ professional psychological help-seeking behavior in a health information search scenario. The following hypothesis was proposed:

**H0:**  *Participants in the no-explanation condition will report significantly lower levels of professional psychological help-seeking behavior compared to those in the explanation condition.*

### 3.1. Method

#### 3.1.1. Design

The study employed a one-factor between-subjects design. The independent variable was the type of explanation (no-explanation vs. with explanation), and the dependent variable was professional psychological help-seeking behavior. The controlled variables were: help-seeking intention, prior help-seeking experience, mental health status, AI usage experience, and attitudes toward AI.

#### 3.1.2. Participants

Given the relevance of the study to XAI and professional psychological help-seeking behavior, participants with academic backgrounds in psychology, psychiatry, medicine, or computer science were excluded. A total of 180 participants were recruited. After excluding careless or invalid responses, 168 valid responses were retained (*M_age_* = 24.89, *SD* = 4.94). The participants were randomly assigned to one of the two explanation types for the experiment (sample characteristics are shown in Table 1).

#### 3.1.3. Materials

Given the sensitive nature of mental health issues, existing studies often employ simulated scenarios where participants are asked to imagine themselves in specific situations to measure help-seeking behaviors (Suka et al., 2016; Lemma et al., 2022; Pimenta et al., 2023). Accordingly, this study adopts a scenario-based simulation approach to measure professional psychological help-seeking behavior.

(1) Mental Health Search Case: To reflect real-world psychological health search scenarios, this study constructed a case based on symptoms of moderate depression and moderate anxiety, which are of current national concern. Symptom descriptions were derived from the Diagnostic and Statistical Manual of Mental Disorders (DSM-5) (Cooper, 2018) and supplemented by real experiences from individuals previously diagnosed with moderate depression and anxiety in interviews. The case narrative includes only the duration of symptoms, psychological symptoms, and physical symptoms. Two doctoral students in psychology were invited to review the case material and provide content revision suggestions. The final case is presented in Appendix A, Table A1.

(2) Explanatory Information: To ensure that the AI-generated explanatory responses met diverse user information needs, a semi-structured interview was conducted with 12 participants before the experiment. Guided by the Health Belief Model, the interview focused on two core questions: “What kind of explanatory information from AI would motivate you to seek professional psychological help?” “What information needs do you expect AI to meet during explanation?” Participants were asked to describe their experiences and thoughts during mental health information searches. The research team conducted a qualitative analysis of the interview data using NVivo 11.0 software.

The coding process included three phases: Open coding: Interview data were broken into meaningful units and assigned codes, resulting in 303 nodes. Focused coding: Codes were grouped and abstracted into 33 focused codes. Axial coding: Focused codes were further refined into 12 axial codes, which were ultimately synthesized into 5 theoretical categories. Based on the standards of Pappas and Woodside (2021), the frequency of each axial and focused code across cases was calculated to determine its representativeness and generalizability (see Appendix A Table A2 for coding results).

Results showed that participants preferred explanatory information directly related to the diagnosis, assessment, and treatment of their mental health concerns when using AI. Specifically, they emphasized the following types of information: Susceptibility information: concrete, personalized symptom descriptions, and matching. Severity information: impact on daily life and potential harm. Effectiveness information: authority of platforms and healthcare professionals, credibility of treatment, and public evaluations. Accessibility information: social support, privacy protection, and cost of treatment, with social security and privacy being of greater concern. Participants also expressed reliance on authoritative data sources and preferred detailed, guidance-oriented explanations rather than simplistic conclusions.

Based on the interview results, this study compiled explanatory information. Since the search engine used in this study is an AI-based search engine, ChatGPT-4.0 was employed to generate the initial experimental materials in order to accurately reflect the real responses of AI in mental health search cases. First, ChatGPT-4.0 was prompted with the query: “I have experienced the following symptoms recently: Over the past month, I have noticed that my sleep quality has been getting worse… Can you analyze what might be wrong with me?” to analyze a mental health search case and obtain the initial response for the non-explanatory group. Then, based on the interview analysis results, ChatGPT-4.0 was prompted with: “Please explain your previous analysis from four aspects: Susceptibility (symptom interpretation and matching), Severity (current seriousness and potential risks), Effectiveness (platform features, professional qualifications, and public reviews), Accessibility (social resources and privacy protections).” This was used to obtain the initial response for the explanatory group. These AI-generated explanations were then refined based on: DSM-5 descriptions (Cooper, 2018) for susceptibility and severity, Materials from X. Chen and Huang (2023) for effectiveness, and interview results for accessibility. Finally, four doctoral and three master’s students in psychology reviewed the content and provided revision suggestions. The final explanatory content was revised and formatted accordingly (see Appendix A Table A3).

**Figure 1:**
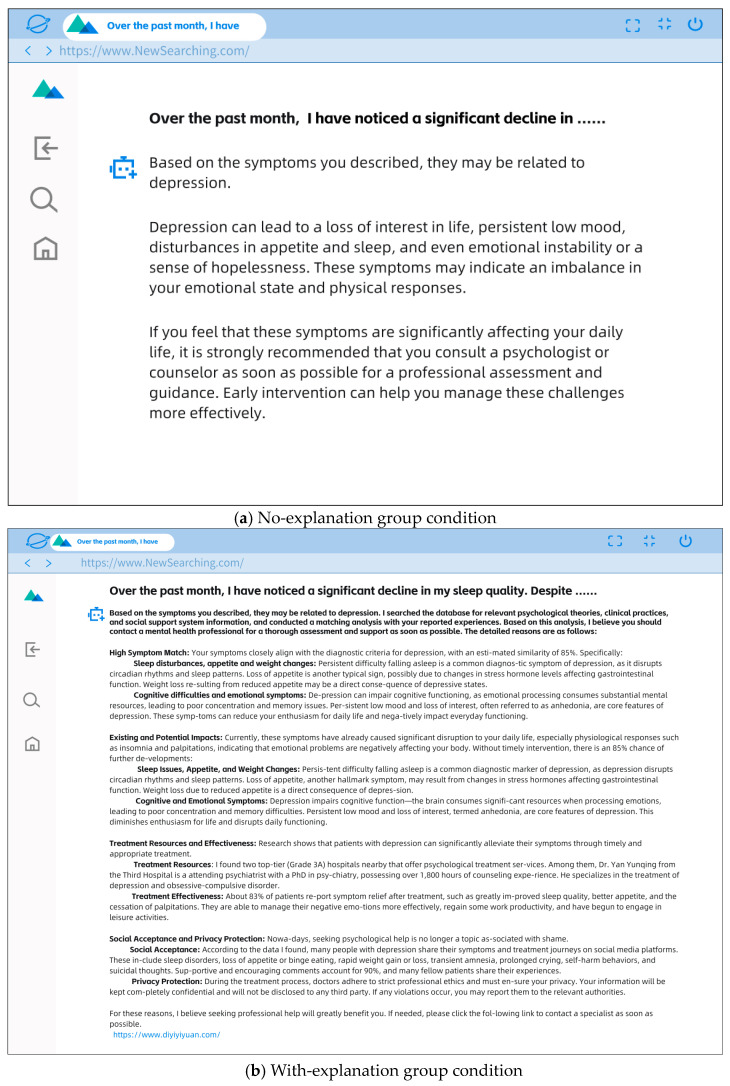
No-explanation (**a**) and With-explanation group (**b**).

To enhance participants’ immersion in the experimental scenario, the presentation was modeled after the interface of AI search engines such as Mita AI, and the final textual content was rendered as images for use in the experiment (see Figure 1).

#### 3.1.4. Procedure

The experiment was conducted using the Sojump platform and comprised three sequential stages. First, participants completed a set of questionnaires designed to measure control variables, including willingness to seek professional psychological help, previous professional help-seeking experience, mental health status, AI experience, and attitude toward AI.

Second, they were randomly assigned to one of two experimental conditions. At the beginning of the experiment, participants were shown the following instructions: “*In this experiment, you will take the role of a user experiencing certain personal difficulties and seeking to understand your condition through an AI search engine. The experiment includes two scenarios, each consisting of a brief context description and a screenshot of an AI-generated search result. Please read both the scenario and the screenshot carefully before answering the following questions. Respond based on your genuine thoughts and feelings in relation to the experimental context.*” Then, participants read a brief scenario involving a mental health context, followed by an AI-generated feedback message tailored to their assigned condition. To ensure the effectiveness of the explainability manipulation, participants’ perceived explainability of the AI system was measured.

After viewing the AI-generated search results, participants were presented with the following definition of explainable AI: “*Explainable AI refers to intelligent systems that can provide clear and transparent information about their decision-making processes, helping users understand how the system processes information and makes recommendations.*” Participants were then asked to rate their agreement with the following three items adapted from Barredo Arrieta et al. (2020) to assess perceived explainability: “The AI system shown in the image is an explainable AI.” “The AI system helps me better understand its information analysis logic.” “The AI system provides more details and reasons to explain its recommendations.” All items were rated on a 7-point Likert scale, from 1 (strongly disagree) to 7 (strongly agree). Since each participant read two scenarios (moderate depression and moderate anxiety), the manipulation check was conducted twice. The final perceived explainability score was calculated as the average of the two measurements. For this scale, the Cronbach’s α coefficients for the two measurements were 0.846 and 0.740; the McDonald’s ω coefficients were 0.847 and 0.742.

Third, participants completed a simulated decision-making task to assess the dependent variable. The estimated experiment duration was between 15 and 20 min, with variation depending on participants’ reading and response time. Upon completion of the experiment, participants will receive compensation of 15 RMB.

#### 3.1.5. Measurements

Professional psychological help-seeking intention: It was measured using the scale developed by Gao and Li (2013). Participants were asked directly how willing they would be to seek professional mental health services if faced with psychological problems they could not resolve on their own. A 7-point Likert scale was used, ranging from 1 (very unwilling) to 7 (very willing), with higher scores indicating greater willingness to seek help.

Past professional help-seeking experience: Participants were asked whether they had ever sought help from professionals (psychiatrists or psychological counselors) through hotlines, the internet, or face-to-face channels for psychological or mental issues. This was a forced-choice (Yes/No) item. A response of “Yes” was scored as 1 (indicating prior help-seeking), and “No” was scored as 0.

Mental health status: Measured using the PHQ-9 (Patient Health Questionnaire) for depression and GAD-7 (Generalized Anxiety Disorder Scale) for anxiety. Each item was rated on a 0–3 scale. Higher total scores indicated poorer mental health status. In this study, the scale demonstrated excellent internal consistency, with Cronbach’s α and McDonald’s ω were both 0.964.

Experience with AI tools: Participants were asked whether they had used generative AI tools. This was a forced-choice (Yes/No) item. “Yes” was scored as 1, and “No” as 0.

Attitudes toward AI: Assessed using the Attitude toward Artificial Intelligence Scale (AIAS-4) developed by Grassini (2023). The scale consists of 4 items rated on a 7-point Likert scale, from 1 (strongly disagree) to 7 (strongly agree). Higher scores indicated a more positive attitude toward AI. In this study, the scale demonstrated excellent internal consistency, with Cronbach’s α and McDonald’s ω were both 0.852.

**Figure A1:**
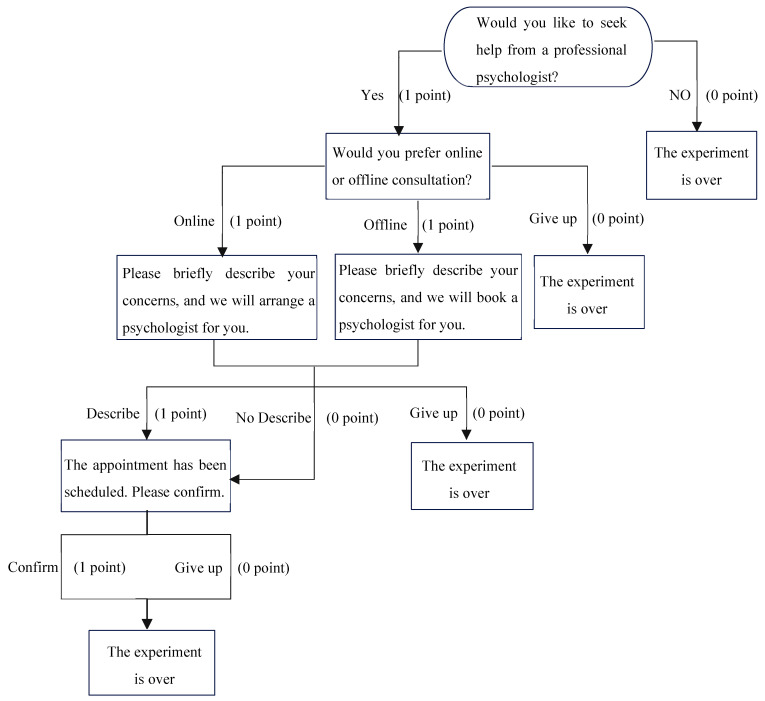
Scoring Method for Professional Psychological Help-Seeking Behavior.

Professional psychological help-seeking behavior: Consistent with prior research that often uses simulated decision-making scenarios to assess behavioral intentions (Cao & Wang, 2019; X. Zhang, 2012; Leichtmann et al., 2023), this study employed a simulated help-seeking task to objectively measure participants’ professional psychological help-seeking behavior. After viewing the AI-generated search response, participants were invited to engage in a simulated appointment process for mental health services. During this simulation, participants were asked to respond to four questions: (1) Would you like to seek help from a professional psychologist? (2) Would you prefer online or offline consultation? (3) Please briefly describe your concerns, and we will arrange/book a psychologist for you. (4) The appointment has been scheduled. Please confirm. Based on the participants’ responses, a behavioral score ranging from 0 to 4 was assigned. Higher scores reflected a stronger behavioral intention to seek professional help. (For detailed scoring rules, see Appendix A, Figure A1) As each participant completed this process for two different cases, the help-seeking behavior was measured twice, and the average score across the two cases was used as the final indicator.

### 3.2. Results

Data were analyzed using SPSS 27.0, with a significance level set at *p* = 0.05. First, a manipulation check was conducted. An independent samples *t*-test was performed with explanation condition (No-explanation vs. With-explanation) as the independent variable and perceived explainability score as the dependent variable. The results showed a significant difference between the two groups on perceived explainability scores. Participants in the no-explanation condition reported significantly lower perceived explainability than those in the explanation condition, indicating the effectiveness of the experimental manipulation (see Table 2).

Next, preliminary analyses were conducted using independent samples *t*-tests. Gender (male vs. female) and employment status (employed vs. student) were treated as independent variables, and professional psychological help-seeking behavior scores as the dependent variable to examine whether there were group differences based on these demographic variables. Additionally, explanation condition (no-explanation vs. with-explanation) was used as the independent variable, with help-seeking intention, help-seeking history, mental health status, and AI usage experience as dependent variables, to assess whether the control variables differed across conditions.

Results indicated that there were no significant differences in professional psychological help-seeking behavior based on gender or occupational status (*p* > 0.05). Furthermore, no significant differences were found between conditions on any of the control variables (*p* > 0.05). Therefore, these variables were not included in subsequent analyses.

**Figure 2:**
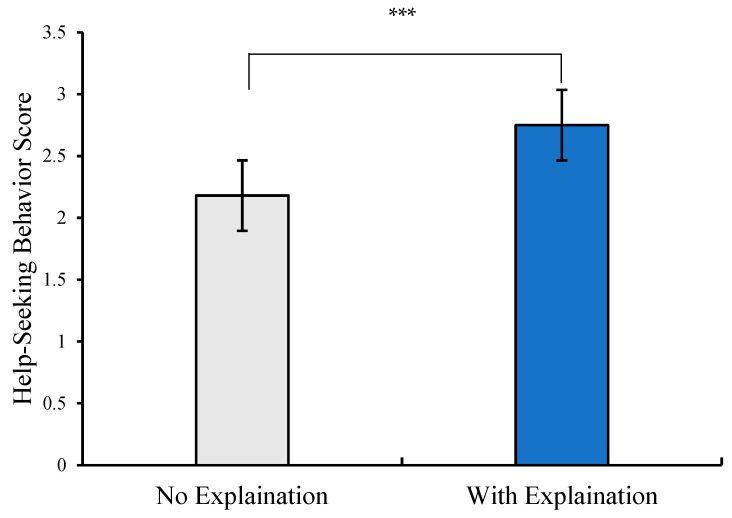
The score differences in Professional Psychological Help-Seeking Behavior(*** *p* < 0.001).

Since study 1 employed a single-factor, two-level randomized group design and confirmed the absence of significant differences in demographic and control variables between the two groups, an independent samples *t*-test was conducted to test the hypothesis. The test used explanation type (no-explanation group vs. with-explanation group) as the independent variable and professional psychological help-seeking behavior score as the dependent variable. The results showed a significant difference between groups. Participants in the no-explanation condition had significantly lower help-seeking behavior scores than those in the explanation condition (see Table 3 and Figure 2).

## 4. Study 2

This experiment aimed to examine the effects of explainable artificial intelligence (XAI) in health information search scenarios on individuals’ cognitive recognition of mental health issues, and to explore whether such recognition mediates the relationship between XAI and professional psychological help-seeking behavior.

The following hypotheses were proposed:

**H1:**  *Mental health literacy has a significant main effect;*

**H1a:**  *Participants with low literacy will report significantly lower recognition of mental health problems compared to those with high literacy;*

**H1b:**  *Participants with low literacy will report significantly lower professional psychological help-seeking behavior scores compared to those with high literacy.*

**H2:**  *Explanation type has a significant main effect;*

**H2a:**  *Participants in the no-explanation condition will show significantly lower recognition of mental health problems than those in the explanation condition;*

**H2b:**  *Participants in the no-explanation condition will show significantly lower help-seeking behavior scores than those in the explanation condition.*

**H3:**  *There is a significant interaction effect between mental health literacy and explanation type, such that the effects of explanation type on psychological problem cognition and help-seeking behavior vary across literacy levels.*

**H4:**  *Psychological problem cognition mediates the effect of XAI and mental health literacy on professional psychological help-seeking behavior.*

### 4.1. Method

#### 4.1.1. Design

A 2 (Mental Health Literacy: low vs. high) × 2 (Explanation Type: no- explanation vs. with-explanation) between-subjects factorial design was employed. Dependent variables conclude with psychological problem cognition and professional psychological help-seeking behavior. Control variables were the same as in Experiment 1.

#### 4.1.2. Participants

To maintain the validity of the experiment, participants with academic backgrounds in psychology, psychiatry, medicine, or computer science were excluded. A total of 200 participants were recruited and randomly assigned to one of the four conditions. After removing inattentive or invalid responses, the final valid sample consisted of 178 participants (*M_age_* = 22.68, *SD* = 2.89). The participants were completely randomly assigned to one of the four experimental conditions (sample characteristics are shown in Table 4).

#### 4.1.3. Experimental Materials

The Mental Health Search Case and Explanatory Information in this experiment were the same as those used in Study 1.

**Figure A2:**
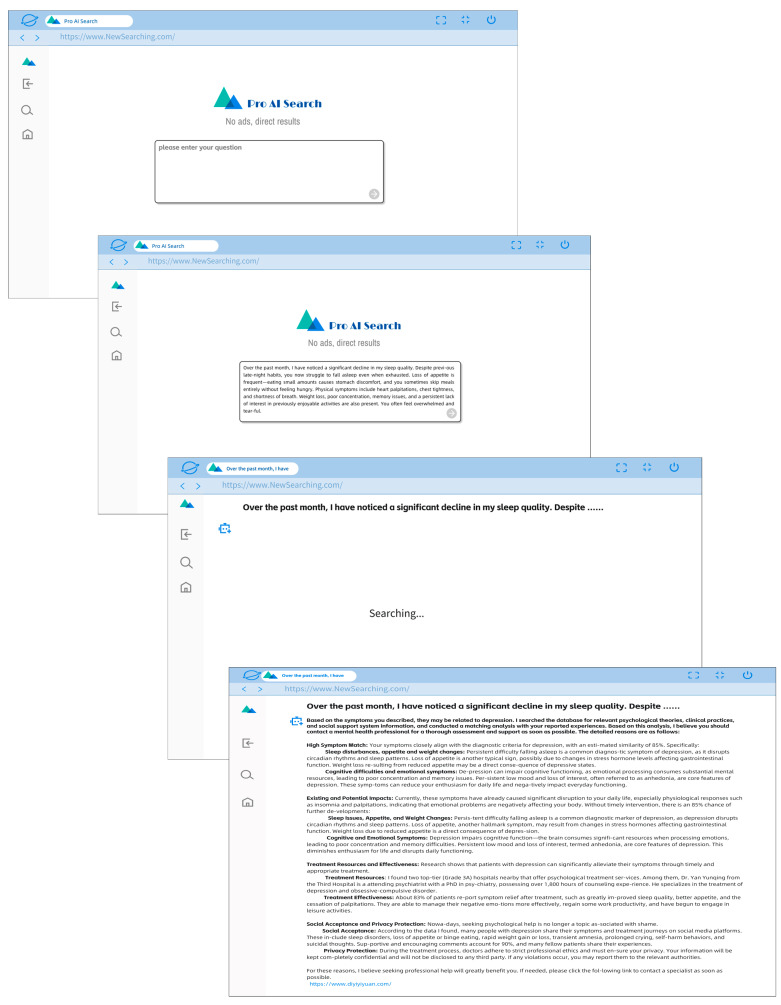
Simulated effect of the search process.

AI Search Engine Simulator: To immerse participants in a realistic AI-powered search scenario from everyday life, this study developed a simulated AI search engine program modeled on the interface and interaction patterns of the well-established, widely used Chinese AI search engine product Metaso AI Search. The simulator replicates the conversational flow of an AI search engine and the word-by-word rendering of text. The resulting search experience is illustrated in Appendix A Figure A2.

#### 4.1.4. Procedure and Measurements

The experiment followed a similar procedure to Study 1. The key difference was that mental health literacy was measured at the beginning. The experiment involved reading mental health-related search cases, reading explanatory information, and a manipulation check. Measurements of the dependent variable were expanded to include assessments of (a) psychological problem cognition and (b) a simulated appointment process for professional psychological help-seeking behavior.

Mental Health Literacy: Assessed using the Mental Health Literacy Scale developed by O’Connor and Casey (2015) and revised by J. J. Liu (2020), comprising 22 items. Scored on a 7-point Likert scale (1 = strongly disagree, 7 = strongly agree). Higher scores indicate better mental health literacy. In this study, the scale demonstrated excellent internal consistency, with Cronbach’s α = 0.785 and McDonald’s ω = 0.897. Confirmatory factor analysis indicated good model fit: χ^2^ (203) = 206, *p* = 0.004, CFI = 0.982, TLI = 0.980, RMSEA = 0.040, 90% CI = [0.023, 0.053], supporting the structural validity of the scale. Participants were categorized into high and low literacy groups based on the median split (Dai & Zhang, 2021).

Psychological Problem Cognition: Measured based on the approaches of Saleeby (2000), Mo and Mak (2009) and J. Yang et al. (2023), comprising 14 items. Scored on a 7-point Likert scale (1 = strongly disagree, 7 = strongly agree). Higher scores reflect better recognition of mental health problems. As each participant read two cases and explanations, the measure was taken twice, and the final score was the average. For this scale, the Cronbach’s α and McDonald’s ω coefficients for the two measurements were both 0.964 and 0.910. Confirmatory factor analysis indicated good model fit: χ^2^ (71) = 75.1, *p* = 0.347, CFI = 0.996, TLI = 0.994, RMSEA = 0.018, 90% CI = [0.000, 0.048], supporting the structural validity of the scale.

**Figure A3:**
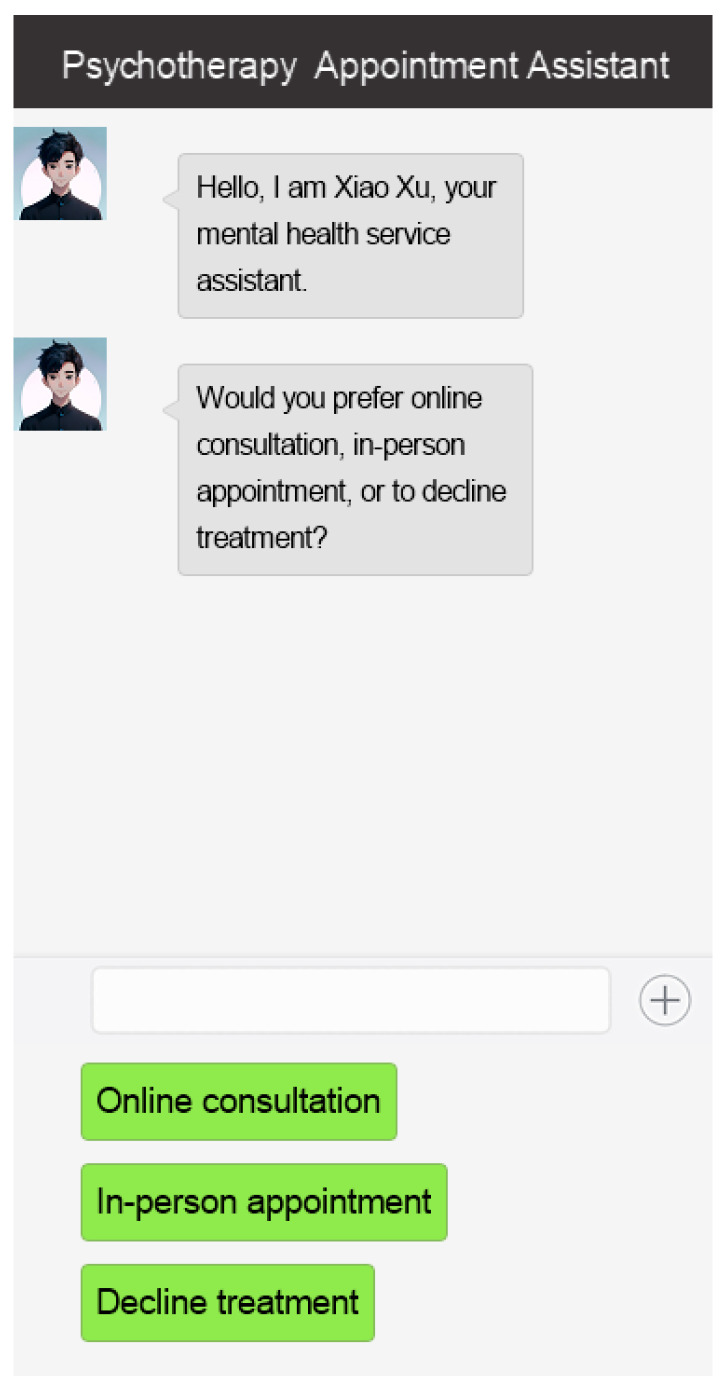
Simulated effect of professional psychological help-seeking service appointment.

Professional psychological help-seeking behavior: The same items as in Study 1 were used. To increase participants’ sense of immersion in the actual appointment procedure, the AI-search-engine simulator presented a dialogue-based mock booking system for professional psychological services; every choice made by the participant was recorded by the program (see Appendix A Figure A3).

### 4.2. Results

#### 4.2.1. Difference Tests

All data analyses were conducted using SPSS version 27.0, with the significance level set at *p* = 0.05. First, manipulation checks were performed: An independent samples *t*-test was conducted with explanation type (No-explanation vs. With-explanation) as the independent variable and perceived explainability as the dependent variable.

Results revealed a significant difference between the two groups. Participants in the no-explanation group perceived significantly lower explainability than those in the explanation group, indicating successful experimental manipulation (see Table 5).

A second independent samples *t*-test tested the manipulation of mental health literacy, using literacy level (low vs. high) as the independent variable and the mental health literacy score as the dependent variable. The result showed a significant difference; the low-literacy group scored significantly lower than the high-literacy group, confirming the effectiveness of this manipulation (see Table 6).

Next, preliminary checks were conducted: Independent samples *t*-tests were used to assess whether gender (male vs. female) and employment status (employed vs. student) had significant effects on psychological problem cognition and professional psychological help-seeking behavior.

Results showed no significant differences (*p* > 0.05), suggesting these demographic variables did not confound the main outcomes. Additional *t*-tests were conducted to examine whether the control variables—help-seeking intention, past help-seeking experience, mental health status, experience with AI, and attitude toward AI—varied across levels of explanation type and mental health literacy. Results indicated significant differences in mental health literacy (see Table 7). No significant differences were found for the other control variables (*p* > 0.05). Therefore, AI experience, help-seeking intention, and mental health status were included as covariates in subsequent analyses.

**Figure 3:**
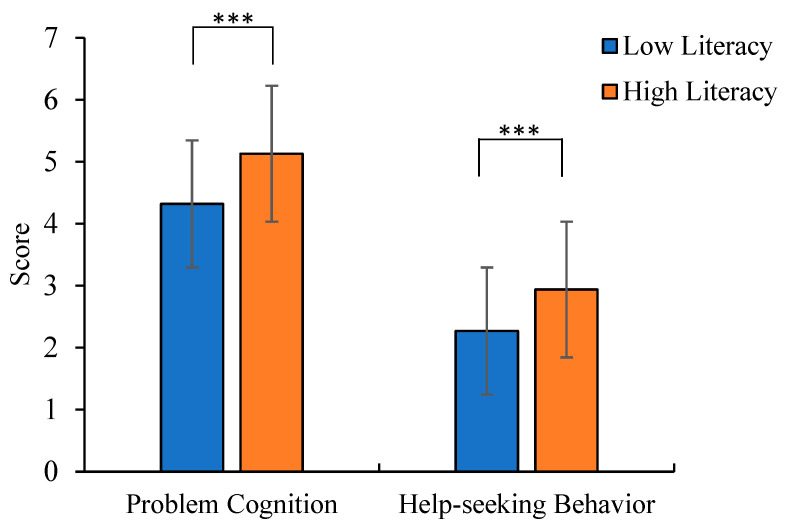
The score differences in problem cognition and help-seeking behavior across mental health literacy levels (*** *p* < 0.001).

As Study 2 utilized a two-factor, four-level randomized group design, a multivariate analysis of covariance (MANCOVA) was performed to examine the main and interaction effects of explanation type and mental health literacy on psychological problem cognition and help-seeking behavior, while controlling for covariates that showed significant differences (AI experience, help-seeking intention, and mental health status). The results showed that after controlling for covariates, the main effect of mental health literacy was significant (see Table 8). Pairwise comparison results indicated significant differences between the two groups in scores of psychological problem cognition and professional psychological help-seeking behavior, with the low literacy group scoring significantly lower than the high literacy group (see Table 8, Figure 3).

**Figure 4:**
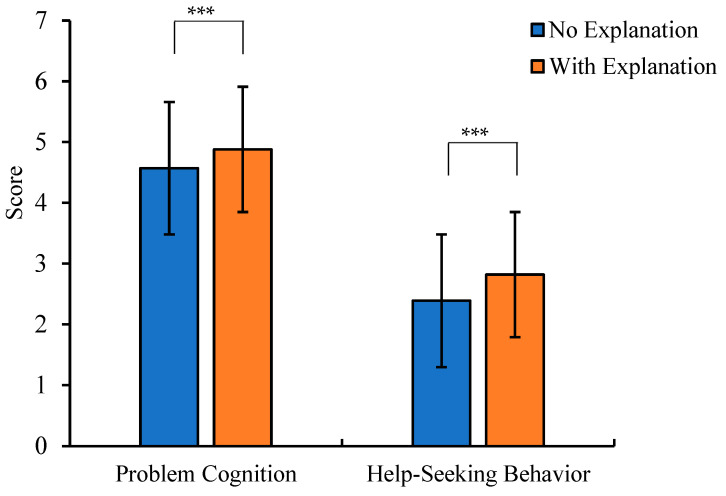
The score differences in problem cognition and help-seeking behavior across different types of explanations (*** *p* < 0.001).

The main effect of explanation type was significant (see Table 9). Pairwise comparison results indicated significant differences between the two groups in scores of psychological problem cognition and professional psychological help-seeking behavior, with the low literacy group scoring significantly lower than the high literacy group (see Table 9, Figure 4).

**Figure 5:**
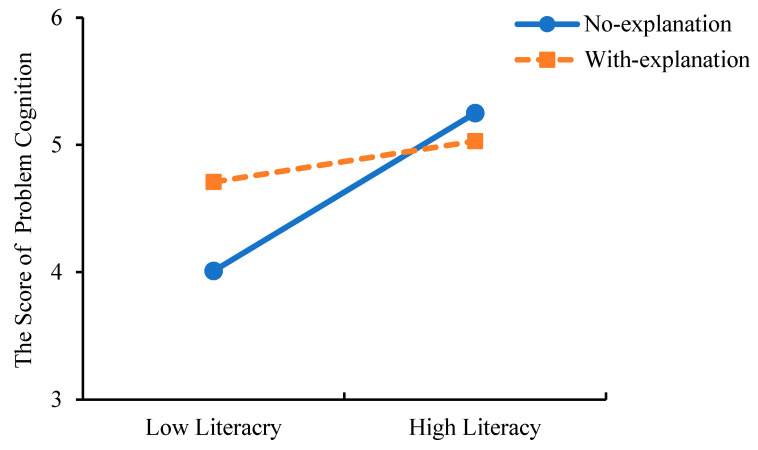
The score differences in problem cognition between explanation types across mental health literacy levels.

The interaction between mental health literacy and explanation type was significant (*F*_(mental issue recognition)_ = 8.077, *p* = 0.005, *η_p_*^2^ = 0.045; *F*_(help-seeking behavior)_ = 6.020, *p* = 0.015, *η_p_*^2^ = 0.034). Simple effect analysis was conducted to examine the interaction effects. The results showed that for psychological problem cognition, in the low literacy group, the no-explanation group scored significantly lower than the explanation group; whereas in the high literacy group, there was no significant difference based on explanation type (see Table 10, Figure 5).

**Figure 6:**
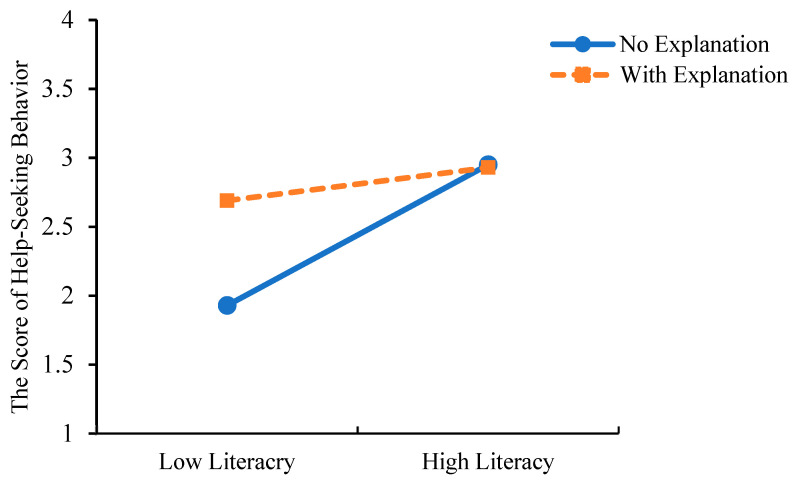
The score differences in help-seeking behavior between explanation types across mental health literacy levels.

For professional psychological help-seeking behavior, in the low literacy group, the no-explanation group scored significantly lower than the explanation group; whereas in the high literacy group, there was no significant difference based on explanation type (see Table 11, Figure 6). The results demonstrate that for individuals with low mental health literacy, AI search engines that provide explanations of their results can more effectively help them recognize their psychological problem and are more likely to encourage professional help-seeking behavior.

#### 4.2.2. Mediation Analysis

To examine the mediating role of psychological problem cognition, Model 4 of PROCESS v4.0 was employed. A bootstrapping procedure with 5000 resamples and a 95% confidence interval (CI) was used. The interaction term (mental health literacy × explanation type) was entered as the independent variable, professional psychological help-seeking behavior as the dependent variable, and psychological problem cognition as the mediating variable. Based on the preliminary analysis, AI experience, help-seeking intention, and mental health status were included as control variables due to their significant effects.

The regression analysis results (see Table 12) showed that: the interaction between mental health literacy and explanation type significantly and positively predicted psychological problem cognition. The interaction also significantly and positively predicted professional psychological help-seeking behavior. Psychological problem cognition significantly and positively predicted professional psychological help-seeking behavior.

**Figure 7:**
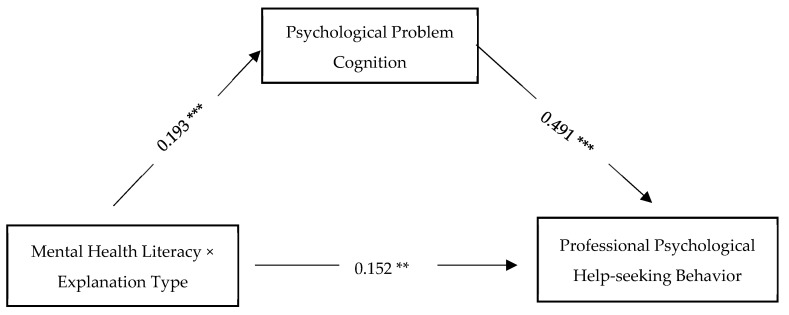
Mediation Effect(** *p* < 0.01, *** *p* < 0.001).

The indirect effect of the interaction on professional psychological help-seeking behavior via psychological problem cognition was 0.095, with a 95% confidence interval of [0.040, 0.166], which does not include zero. This indicates that the mediation effect of psychological problem cognition is statistically significant (see Table 13 and Figure 7).

## 5. Discussion

### 5.1. The Effect of XAI on Professional Psychological Help-Seeking Behavior

Study 1 employed a scenario-based imagination experiment to investigate the effect of XAI on individuals’ professional psychological help-seeking behavior. The results revealed that participants in the explanation condition scored significantly higher on professional psychological help-seeking behavior than those in the no-explanation condition, indicating that XAI can effectively promote individuals’ willingness to seek professional psychological help. This finding supports the hypothesis of Study 1 and aligns with previous research (Leichtmann et al., 2023; Markus et al., 2021). Prior studies have demonstrated that enhancing the transparency of AI decisions by explaining the underlying rationale can effectively improve individual decision-making in high-risk domains such as medical decision-making and toxic mushroom identification. The present study extends these findings to the mental health domain, suggesting that similar effects apply in psychologically sensitive contexts.

Most advanced machine learning models exhibit the characteristics of a “black box”, meaning that the decision-making process between input and output is often opaque and difficult to interpret (S. Y. Zhang & Pi, 2023). As a result, individuals tend to adopt a conservative attitude toward AI recommendations, particularly when decisions involve high-stakes areas such as healthcare or financial investment. However, explainability in AI systems can effectively alleviate this cautious attitude. Research has shown that when information systems provide process-level information that is easy to comprehend, individuals are more likely to engage with the system and report increased levels of trust (Shin & Park, 2019). By offering decision rationales, XAI enhances users’ trust in AI, thereby reducing psychological resistance. Such trust is especially crucial in domains involving personal privacy, such as mental health, where trust is a key determinant of individuals’ willingness to seek professional help (Zhao et al., 2011). XAI improves transparency by disclosing its decision logic, thereby reducing users’ concerns about misjudgment and alleviating the fear of unpredictability typically associated with black-box models. Furthermore, according to the “Stage-Process Model” of help-seeking, individuals in the self-assessment stage must weigh the severity of their problem against the reliability of external resources (Jiang & Xia, 2006). The explanation mechanism provided by AI enhances the perceived reliability of the system, enabling users to move more swiftly into the “help-seeking evaluation” stage.

### 5.2. XAI and Mental Health Literacy on Psychological Problem Cognition and Professional Psychological Help-Seeking Behavior

Study 2 examined the effects of XAI and mental health literacy on individuals’ psychological problem cognition and professional psychological help-seeking behavior, and tested the mediating role of psychological problem cognition. the results revealed the following: (1) XAI significantly improved both psychological problem cognition and professional psychological help-seeking behavior, with participants in the explanation condition scoring significantly higher on both measures than those in the non-explanation condition. (2) Higher mental health literacy was associated with stronger psychological problem cognition and greater professional help-seeking, with high-literacy individuals outperforming their low-literacy counterparts. (3) There was a significant interaction effect between mental health literacy and XAI. Among participants with low mental health literacy, those in the explanation condition scored significantly higher on both outcome variables than those in the no-explanation condition. However, among individuals with high mental health literacy, this difference was not significant. These findings indicate that XAI can effectively enhance both psychological problem cognition and help-seeking behavior, especially among individuals with low levels of mental health literacy.

Barredo Arrieta et al. (2020) have argued that XAI enhances trust and user acceptance by providing transparent and interpretable decision-making processes. In the mental health context, such transparency is particularly critical, as individuals need to understand AI-generated suggestions to accurately assess their psychological state (Graham et al., 2019). This effect is especially pronounced among those with low mental health literacy, who lack sufficient knowledge and therefore rely more on external informational cues to interpret their condition. When XAI provides explanatory information, such as symptom matching, the degree of fit, current implications, and projected symptom development, users are better able to comprehend the AI’s judgment, which in turn enhances perceived usefulness and ease of use. According to the Technology Acceptance Model (Venkatesh & Davis, 2000), when individuals perceive a technology as useful and easy to use, they are more likely to adopt and trust it. This trust in AI promotes deeper information processing, leading to greater awareness and understanding of one’s mental health status. previous research has shown that exposure to mental health-related media content can improve individuals’ awareness of their psychological issues (X. Song et al., 2024). Similarly, XAI can help individuals, especially those lacking prior mental health knowledge, understand the urgency and importance of their condition, thereby facilitating better recognition and help-seeking behavior. However, for individuals with high mental health literacy, who already possess adequate knowledge and can independently assess their condition, the additional explanatory support provided by XAI offers limited incremental benefits. This interaction effect highlights that the influence of XAI is especially pronounced among individuals with lower mental health literacy, where it effectively compensates for gaps in their understanding and promotes help-seeking.

Furthermore, our analyses indicated no significant differences in the cognition of psychological problems across demographic characteristics such as gender and employment status. This finding contrasts with previous studies in mental health awareness (Chu et al., 2025). One possible reason is that the scenario-based tasks directed participants’ s attention to concrete details of the situation, thereby reducing the influence of background characteristics (Goel et al., 2024). Although the results differ from prior literature, they suggest that the benefits of XAI in enhancing mental health recognition may be broadly applicable across diverse demographic groups.

### 5.3. The Mediating Role of Psychological Problem Cognition

Study 2 also examined the mediating role of psychological problem cognition in the effects of XAI and mental health literacy on professional psychological help-seeking behavior. The results showed that psychological problem cognition mediated the effects of both mental health literacy and XAI on professional psychological help-seeking behavior, suggesting that XAI’s positive influence on help-seeking was fully realized through enhancing individuals’ recognition of mental health problems. research has shown that personalized interventions can significantly enhance individuals’ recognition of mental health problems (Kazdin & Blase, 2011). Explainable AI (XAI), by analyzing individual data, can offer customized recommendations and explain why taking action is both effective and free from unnecessary concerns. However, the impact of such personalization is also moderated by individuals’ level of mental health literacy. Specifically, individuals with lower mental health literacy are more reliant on the personalized information provided by XAI, whereas those with higher literacy may have less need for such supplementary information due to their pre-existing knowledge base. This moderating effect is also supported by (J. Yang et al., 2023), who found that the information individuals receive significantly influences their health beliefs regarding mental health issues, with perceived benefits being particularly important.

In the current study, XAI provided participants with locally accessible, reliable mental health resources, evaluations of service quality, and social attitudes toward mental health, helping users recognize that professional mental health services are both approachable and trustworthy (Hofmann et al., 2009). This, in turn, enhanced participants’ perceptions of the effectiveness and accessibility of mental health interventions. For individuals with low mental health literacy, who tend to lack awareness of the availability and effectiveness of professional psychological services, XAI’s explanatory capabilities help fill these cognitive gaps by highlighting the benefits of mental health interventions.

According to the Health Belief Model, which posits that cognition drives behavior, improved awareness fosters greater motivation to engage in health-promoting actions, such as seeking professional help. A growing body of research supports the idea that individuals’ recognition of their mental health status is a key factor influencing their help-seeking attitudes, behavioral intentions, and actual behaviors (Billings et al., 2021; F. L. Li et al., 2016; Gong & Song, 2024). By providing evidence-based reasoning and tailored advice, XAI enhances users’ confidence in managing their mental health, thereby promoting professional psychological help-seeking behavior. Importantly, this facilitative effect is particularly pronounced among individuals with low mental health literacy, as the explanatory information offered by XAI can directly improve their recognition of psychological problems and increase trust in help-seeking actions. These mechanisms work in concert to establish the mediating role of psychological problem cognition in the relationship between XAI and professional psychological help-seeking behavior.

While our findings highlight the positive effects of XAI on mental health recognition and help-seeking, it is equally important to acknowledge potential risks associated with the use of explainability in sensitive contexts (Martens et al., 2025). Explanations that provide very specific or probabilistic information may unintentionally heighten users’ anxiety or lead them to overinterpret AI outputs, especially when they are already in a vulnerable psychological state (Martens et al., 2025). Moreover, when model confidence information is poorly calibrated, it can distort users’ trust and lead either to inappropriate reliance or to undue skepticism, both of which undermine effective decision-making (Cabitza et al., 2025). Other studies suggest that certain explanatory formats can unintentionally amplify the persuasive force of AI recommendations even when the underlying judgment is inaccurate, which raises concerns about potential misguidance in sensitive settings such as mental health (Danry et al., 2024). Taken together, these findings suggest that explainability should not be assumed to be uniformly beneficial and can introduce new risks when applied inappropriately.

## 6. Strengths and Limitations

This study has both theoretical and practical strengths. Theoretically, building upon previous research, it incorporates information media as a critical external cue to explore the mechanisms through which explainable artificial intelligence (XAI) influences professional psychological help-seeking behavior in the context of health information search. By conducting experiments with individuals possessing varying levels of mental health literacy, the study identifies behavioral differences in response to explainable information. These findings enhance our understanding of the interaction between cognitive and informational factors in professional help-seeking and provide a valuable theoretical basis for advancing intelligent psychological health interventions. Practically, the study highlights the potential of XAI to enhance users’ trust in mental health information and promote help-seeking behavior. The results offer new insights into how explainable AI can influence behavioral decision-making and suggest concrete, evidence-based strategies for applying XAI in the mental health domain. However, the study also has several limitations:

Sample limitations: The study sample was limited to young adults aged 17–38, with a relatively narrow distribution in terms of age, education level, and professional background. This limits the external validity of the findings and reduces their generalizability to other populations. Future studies should include participants from a broader age range (e.g., adolescents, middle-aged adults, and older adults) to examine the effects of AI explainability on professional psychological help-seeking behavior across different demographic groups.

Induction method: The experimental design employed a scenario-based imagination paradigm, presenting participants with mental health-related cases and instructing them to imagine themselves in the described situations. Although we actively measured and statistically controlled for participants’ baseline mental health status using validated scales (e.g., PHQ-9, GAD-7) to help mitigate potential confounding effects of pre-existing psychological distress on reasoning and decision-making processes, a gap with actual mental health conditions remains. Future research could enhance ecological and psychological validity by recruiting participants with clinically relevant mental health experiences and designing personalized scenarios based on their real-life contexts.

Interface realism: The virtual simulation interface used in this study was relatively static and lacked interactivity. Moreover, the experiment was conducted entirely in a controlled laboratory setting, which differs significantly from real-world AI search and interaction environments, reducing ecological validity. Future research should consider optimizing the interface design and interaction modalities, or even developing fully functional AI systems for real-time interaction to increase authenticity and ecological validity.

Measurement of help-seeking behavior: In this study, simulated appointment selection was used as a proxy for professional psychological help-seeking behavior. However, this may not accurately reflect real-world behavior. Future studies should incorporate longitudinal tracking data to capture actual help-seeking behaviors over time. Moreover, it is advisable to include eye-tracking indicators, EEG, and other measures to improve the comprehensiveness and reliability of the behavioral assessments.

Furthermore, while our study focused on the benefits of XAI, future research should directly investigate its potential negative psychological impacts and ethical dilemmas to establish guidelines for its safe implementation.

## 7. Conclusions

This study explored the impact of explainable artificial intelligence (XAI) and mental health literacy on recognition of psychological problems and professional psychological help-seeking behavior. Through two experiments, the findings demonstrated that XAI significantly enhances both psychological problem cognition and help-seeking behavior, especially among individuals with lower mental health literacy. Furthermore, psychological problem cognition was found to mediate the relationship between XAI and professional psychological help-seeking behavior. These results highlight the potential of XAI as a supportive tool in mental health interventions and underscore the importance of tailoring digital health technologies to users’ cognitive characteristics.

## Figures and Tables

**Table 1:** Sample Characteristics of Study 1.

		No-Explanation (*n* = 87)	With-Explanation (*n* = 81)
	*M_age_*	22.44	27.53
	SD	4.22	4.28
Gender			
	Male	47	41
	Female	40	40
Occupational Status			
	Student	74	61
	Employed	13	20

**Table 2:** Manipulation Check Results.

	M (SD)	t	*p*	Cohen’s d	95% CI
No-explanation	4.52 (1.04)	−4.728	<0.001	−0.730	−1.042, −0.416
With-explanation	5.19 (0.77)				

**Table 3:** Differences in Professional Psychological Help-Seeking Behavior Scores.

	M (SD)	t	*p*	Cohen’s d	95% CI
No-explanation	2.18 (1.11)	−3.574	<0.001	−0.552	−0.859, −0.243
With-explanation	2.75 (0.94)				

**Table 4:** Sample Characteristics of Study 2.

		A Group (*n* = 49)	B Groups (*n* = 39)	C Groups (*n* = 41)	D Groups (*n* = 49)
	*M_age_*	22.29	23.46	21.90	23.10
	SD	3.01	3.04	2.47	2.85
Gender					
	Male	25	19	21	27
	Female	24	20	20	22
Occupational Status					
	Student	38	31	35	36
	Employed	11	8	6	13

Notes: A Group = Low Literacy/No-Explanation; B Group = Low Literacy/With-Explanation; C Group = High Literacy/No-Explanation; D Group = High Literacy/With-Explanation.

**Table 5:** Manipulation Check Results.

	M (SD)	t	*p*	Cohen’s d	95% CI
No-explanation	4.62 (1.00)	−3.026	0.003	−0.454	−0.751, −0.155
With-explanation	5.05 (0.87)				

**Table 6:** Manipulation Check Results.

	M (SD)	t	*p*	Cohen’s d	95% CI
No-explanation	3.97 (0.72)	−12.730	<0.001	−1.908	−2.261, −1.551
With-explanation	4.98 (0.22)				

**Table 7:** Tests of Differences in Control Variables Across Levels of Mental Health Literacy.

	t	*p*	Cohen’s d	95% CI
Al experience	−2.491	0.014	−0.373	−0.669, −0.077
Help-seeking intention	−7.209	<0.001	−1.081	−1.394, −0.765
Mental health status	2.594	0.020	0.389	0.092, 0.685

**Table 8:** The score differences in psychological problem cognition and professional psychological help-seeking behavior across mental health literacy levels.

		M (SD)	F	*p*	*η_p_* ^2^
Psychological Problem Cognition	Low Literacy	4.32 (0.86)	19.513	<0.001	0.102
	High Literacy	5.13 (0.58)			
Professional Psychological Help-Seeking Behavior	Low Literacy	2.27 (0.92)	12.603	<0.001	0.069
	High Literacy	2.94 (0.81)			

**Table 9:** The score differences in psychological problem cognition and professional psychological help-seeking behavior across different explanation types.

		M (SD)	F	*p*	*η_p_* ^2^
Psychological Problem Cognition	No-explanation	4.57 (0.86)	7.928	0.005	0.044
	With-explanation	4.88 (0.77)			
Professional Psychological Help-Seeking Behavior	No-explanation	2.39 (0.95)	9.765	0.002	0.054
	With-explanation	2.82 (0.85)			

**Table 10:** The score differences in psychological problem cognition between explanation types across mental health literacy levels.

		M (SD)	F	*p*	*η_p_* ^2^
Low Literacy	No-explanation	4.01 (0.73)	15.922	<0.001	0.120
	With-explanation	4.71 (0.86)			
High Literacy	No-explanation	5.25 (0.42)	0.020	0.889	0.000
	With-explanation	5.03 (0.67)			

**Table 11:** The score differences in professional psychological help-seeking behavior between explanation types across mental health literacy levels.

		M (SD)	F	*p*	*η_p_* ^2^
Low Literacy	No-explanation	1.93 (0.87)	15.392	<0.001	0.083
	With-explanation	2.69 (0.80)			
High Literacy	No-explanation	2.95 (0.72)	0.129	0.719	0.001
	With-explanation	2.93 (0.89)			

**Table 12:** Regression Results of the Mediation Analysis.

Regression		Model Fit Indices			Standardized	t
Outcome Variable	Predictor Variable	R	R^2^	F	β	
Mental Health Problem Cognition	Mental Health Literacy × Explanation Type	0.629	0.396	28.317	0.193	4.469 ***
Professional Psychological Help-Seeking Behavior	Mental Health Literacy × Explanation Type	0.570	0.324	16.517	0.152	2.829 **
	Problem Cognition				0.491	5.467 ***

Notes: ** *p* < 0.01, *** *p* < 0.001.

**Table 13:** Bootstrap Results for the Mediation Path.

Mediation Path	Effect	BootSE	BootLLCI	BootULCI
Total Effect	0.247	0.055	0.138	0.356
Direct Effect	0.152	0.054	0.046	0.259
Indirect Path X -> M -> Y	0.095	0.033	0.040	0.166

Notes: X = Mental Health Literacy × Explanation Type; Y = Professional Psychological Help-Seeking Behavior; M = Psychological Problem Cognition.

## Data Availability

The data presented in this study are available on request from the corresponding author. The data are not publicly available due to privacy.

## Appendix A

**Table A1:** Mental Health Search Cases.

Symptoms	Case Description
Moderate Depression	Over the past month, you have noticed a significant decline in your sleep quality. Despite previous late-night habits, you now struggle to fall asleep even when exhausted. Loss of appetite is frequent—eating small amounts causes stomach discomfort, and you sometimes skip meals entirely without feeling hungry. Physical symptoms include heart palpitations, chest tightness, and shortness of breath. Weight loss, poor concentration, memory issues, and a persistent lack of interest in previously enjoyable activities are also present. You often feel overwhelmed and tearful.
Moderate Anxiety	Over the past month, you have experienced heightened tension and fear, particularly before social interactions or public speaking. Avoidance of social events due to fear of embarrassment is common. Physical symptoms include sweating palms, rapid heartbeat, nausea, dizziness, and difficulty breathing. Gastrointestinal issues like stomach pain and diarrhea often occur before important events. Low self-confidence and feelings of inadequacy in social situations are persistent.

**Table A2:** Interview Results.

Theoretical Coding	Axial Coding	Focus Coding	Representativeness	Example of Core Perspectives
**Susceptibility Information**	1. Symptom Explanation		12G	
		1.1 Professional Terms	8T	Technical terms from search results are acceptable but do not require further explanation.
		1.2 Specific Symptom Descriptions	11G	Detailed descriptions of symptoms, both physical and psychological, that are closely related to daily life.
	2. Symptom Matching		12G	
		2.1 Symptom Checklist	1R	A checklist-like tool to self-screen and identify potential issues.
		2.2 Personalized Answers	6T	Tailored responses are preferred over generic online information.
		2.3 Symptom Match Rate	12G	A quantified symptom match percentage (e.g., 85%) increases confidence in the diagnosis.
**Severity Information**	1. Current Severity		10T	
		1.1 Self-Recovery Difficulty	2R	I first check if I can recover on my own; if not, I seek professional help.
		1.2 Impact on Daily Life	6T	The decision to seek help depends on whether symptoms disrupt normal life.
	2. Potential Harm		11G	
		2.1 Progression Trends	5V	Understanding the condition’s progression and potential risks (e.g., life-threatening consequences).
		2.2 Harm to Others	1R	Worry that emotional instability might negatively affect family members.
		2.3 Harm to Self	8T	Symptoms like social withdrawal or physical deterioration may prompt seeking help.
**Effectiveness Information**	1. Platform Features		11G	
		1.1 Professional Equipment	1R	Trust in platforms with specialized diagnostic tools or validated assessment scales.
		1.2 Credentials	6T	Government or institutional certifications enhance credibility.
		1.3 Platform Security	3V	Secure communication channels with professionals are critical.
		1.4 Treatment Plans	6T	Clear guidance on symptom management and recovery strategies.
	2. Professional Traits		12G	
		2.1 Clinical Experience	10T	Prioritize doctors with relevant case experience and expertise.
		2.2 Qualifications	10T	Educational background, titles, and specialization areas are important.
	3. Public Reviews		11G	
		3.1 Peer Recommendations	1R	Recommendations from trusted acquaintances with similar issues.
		3.2 Objective Descriptions	1R	Objective accounts of treatment processes and outcomes are valued.
		3.3 Treatment Outcomes	6T	Patient testimonials on symptom improvement post-treatment are persuasive.
**Accessibility Information**	1. Treatment Costs		3V	
		1.1 Time Costs	1R	Hospital visits are time-consuming and exhausting.
		1.2 Distance Costs	2R	Geographical barriers reduce willingness to seek offline help.
		1.3 Financial Costs	2R	High costs may deter individuals from seeking help for minor issues.
	2. Social Support		6T	
		2.1 Information Access	3V	Lack of awareness about mental health resources delays help-seeking.
		2.2 Help-Seeking Channels	6T	Limited access to reliable platforms is a barrier.
		2.3 Social Stigma	6T	Fear of judgment or discrimination prevents help-seeking.
	3. Privacy Protection		6T	
		3.1 Confidentiality Obligations	1R	Trust in medical professionals’ ethical duty to protect privacy.
		3.2 Privacy Assurance	6T	Cross-platform data aggregation improves reliability.
**Information Characteristics**	1. Data Sources		10V	
		1.1 Comprehensive Data	3V	Cross-platform data aggregation improves reliability.
		1.2 Professional Data	10V	Information from authoritative sources (e.g., hospital websites, journals).
		1.3 Peer Experiences	5V	Shared experiences from individuals with similar symptoms are helpful.
	2. Content Presentation		8T	
		2.1 Detailed Explanations	5V	In-depth explanations of data sources and matching logic enhance trust.
		2.2 Guidance-Oriented Information	6T	AI should provide actionable recommendations rather than definitive conclusions.
		2.3 Concise Explanations	1R	Brief but evidence-supported explanations are sufficient.

Notes: G = General (11–12 cases); T = Typical (6–10 cases); V = Variant (3–5 cases); R = Rare (1–2 cases).

**Table A3:** Explanatory Information.

Group	Information Dimension	Interview Findings	
**No-explanation**			Based on the symptoms you described, they may be related to depression. Depression can lead to a loss of interest in life, persistent low mood, disturbances in appetite and sleep, and even emotional instability or a sense of hopelessness. These symptoms may indicate an imbalance in your emotional state and physical responses. If you feel that these symptoms are significantly affecting your daily life, it is strongly recommended that you consult a psychologist or counselor as soon as possible for a professional assessment and guidance. Early intervention can help you manage these challenges more effectively.
**With-explanation**			Based on the symptoms you described, they may be related to depression. I searched the database for relevant psychological theories, clinical practices, and social support system information, and conducted a matching analysis with your reported experiences. Based on this analysis, I believe you should contact a mental health professional for thorough assessment and support as soon as possible. The detailed reasons are as follows:
	**Susceptibility Information**	Technical terms Specific symptom description Targeted response Symptom alignment	High Symptom Match: Your symptoms closely align with the diagnostic criteria for depression, with an estimated similarity of 85%. Specifically: Sleep disturbances, appetite and weight changes: Persistent difficulty falling asleep is a common diagnostic symptom of depression, as it disrupts circadian rhythms and sleep patterns. Loss of appetite is another typical sign, possibly due to changes in stress hormone levels affecting gastrointestinal function. Weight loss resulting from reduced appetite may be a direct consequence of depressive states. Cognitive difficulties and emotional symptoms: Depression can impair cognitive functioning, as emotional processing consumes substantial mental resources, leading to poor concentration and memory issues. Persistent low mood and loss of interest, often referred to as anhedonia, are core features of depression. These symptoms can reduce your enthusiasm for daily life and negatively impact everyday functioning.
	**Severity Information**	Severity of physical issues Impact on daily life Development trend Self-harm	Existing and Potential Impacts: Currently, these symptoms have already caused significant disruption to your daily life, especially physiological responses such as insomnia and palpitations, indicating that emotional problems are negatively affecting your body. Without timely intervention, there is an 85% chance of further developments: Sleep Issues, Appetite, and Weight Changes: Persistent difficulty falling asleep is a common diagnostic marker of depression, as depression disrupts circadian rhythms and sleep patterns. Loss of appetite, another hallmark symptom, may result from changes in stress hormones affecting gastrointestinal function. Weight loss due to reduced appetite is a direct consequence of depression. Cognitive and Emotional Symptoms: Depression impairs cognitive function—the brain consumes significant resources when processing emotions, leading to poor concentration and memory difficulties. Persistent low mood and loss of interest, termed anhedonia, are core features of depression. This diminishes enthusiasm for life and disrupts daily functioning.
	**Effectiveness Information**	Professional qualifications Clinical experience Academic degrees, titles, and specialties Treatment effectiveness	Treatment Resources and Effectiveness: Research shows that patients with depression can significantly alleviate their symptoms through timely and appropriate treatment. Treatment Resources: I found two top-tier (Grade 3A) hospitals nearby that offer psychological treatment services. Among them, Dr. Yan Yunqing from the Third Hospital is an attending psychiatrist with a PhD in psychiatry, possessing over 1800 h of counseling experience. He specializes in the treatment of depression and obsessive-compulsive disorder. Treatment Effectiveness: About 83% of patients report symptom relief after treatment, such as greatly improved sleep quality, better appetite, and the cessation of palpitations. They are able to manage their negative emotions more effectively, regain some work productivity, and have begun to engage in leisure activities.
	**Accessibility Information**	Information sources Help-seeking channels Stigmatization Confidentiality commitment	Social Acceptance and Privacy Protection: Nowadays, seeking psychological help is no longer a topic associated with shame. Social Acceptance: According to the data I found, many people with depression share their symptoms and treatment journeys on social media platforms. These include sleep disorders, loss of appetite or binge eating, rapid weight gain or loss, transient amnesia, prolonged crying, self-harm behaviors, and suicidal thoughts. Supportive and encouraging comments account for 90%, and many fellow patients share their experiences. Privacy Protection: During the treatment process, doctors adhere to strict professional ethics and must ensure your privacy. Your information will be kept completely confidential and will not be disclosed to any third party. If any violations occur, you may report them to the relevant authorities. For these reasons, I believe seeking professional help will greatly benefit you. If needed, please click the following link to contact a specialist as soon as possible. https://www.diyiyiyuan.com/.
